# Supplementary figures and images for: Silencing of the 20S proteasomal subunit-α6 triggers full oogenesis arrest and increased mRNA levels of the selective autophagy adaptor protein p62/SQSTM1 in the ovary of the vector Rhodnius prolixus
Source: PLoS Negl Trop Dis. 2023 Jun 2;17(6):e0011380. doi: 10.1371/journal.pntd.0011380 (PMC10266689; doi:10.1371/journal.pntd.0011380)

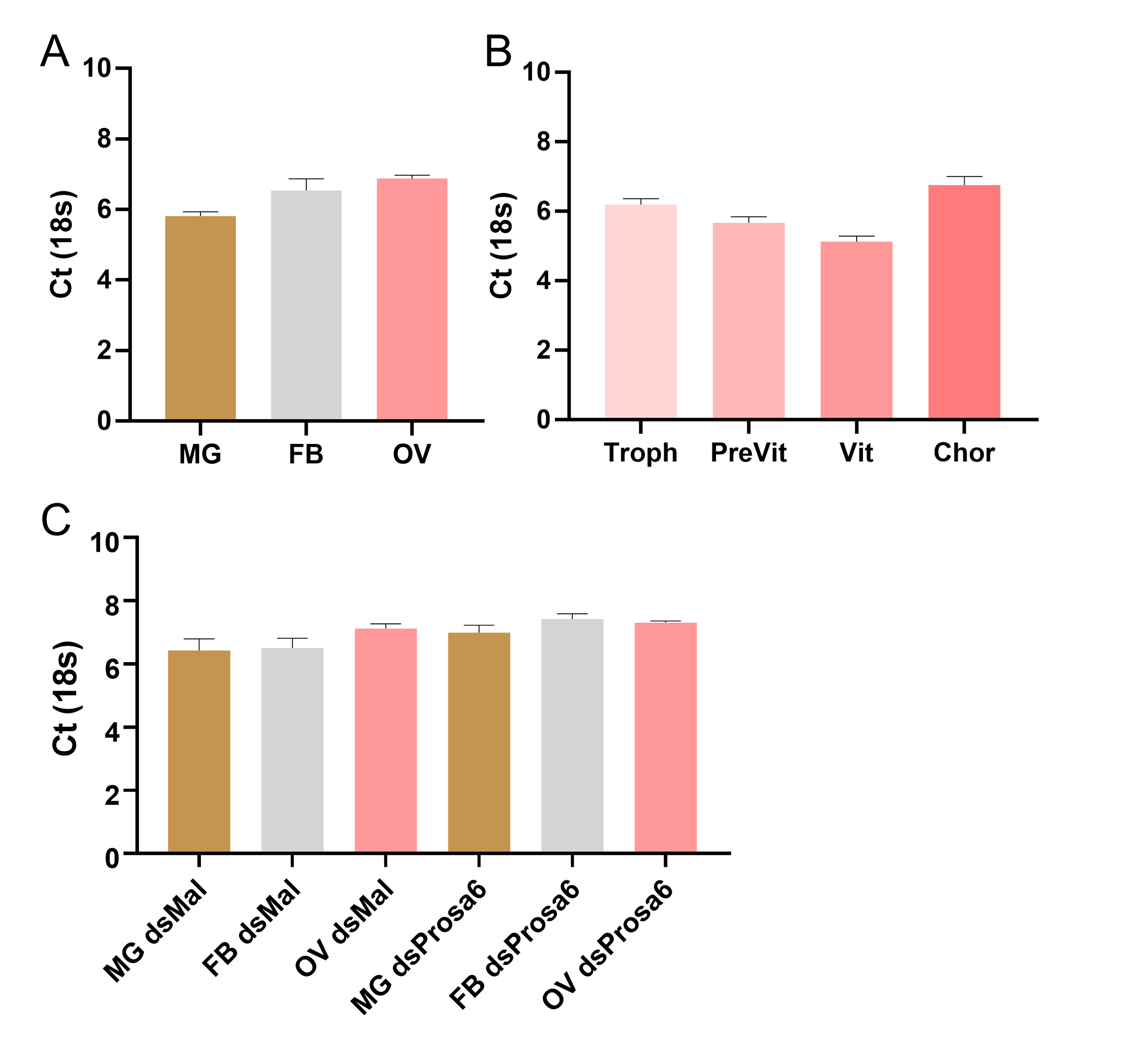

Supplement: S1 Fig — 18s Cts obtained from different samples and conditions. (A) RNA extracted from the ovary, midgut and fat body. (B) RNA extracted from the different parts of the ovariole (troph, tropharium; PV, previtellogenic follicle; Vit, Vitellogenic follicle; Chor, chorionated oocyte). (C) RNA extracted from the ovary and fat body of control (dsMal) and silenced insects. All samples were dissected 7 days after the blood meal. (TIF) [file pntd.0011380.s001.tif]

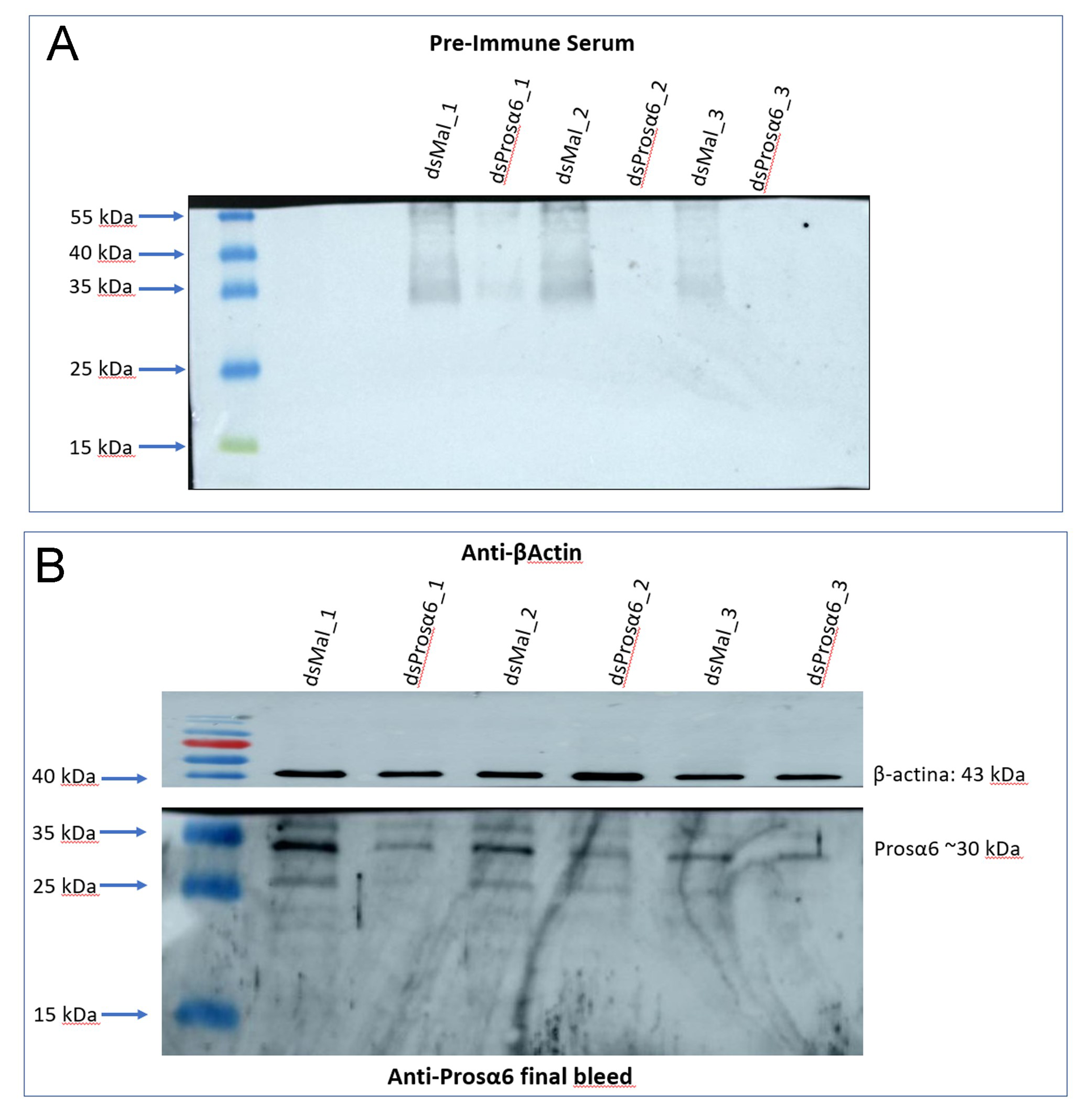

Supplement: S2 Fig — (A) Immunoblotting using the pre-immune serum from the immunized rabbit in control (dsMal) and silenced (dsProsα6) ovary samples. (B) Immunoblotting using the final bleed serum from the immunized rabbit in control (dsMal) and silenced (dsProsα6) ovary samples. All samples were dissected 7 days after the blood meal. A pool of 3 ovaries were used for each experiment (n = 3). (TIF) [file pntd.0011380.s002.tif]
