# Supplementary material for: Silencing of the 20S proteasomal subunit-α6 triggers full oogenesis arrest and increased mRNA levels of the selective autophagy adaptor protein p62/SQSTM1 in the ovary of the vector Rhodnius prolixus
Source: PLoS Negl Trop Dis. 2023 Jun 2;17(6):e0011380. doi: 10.1371/journal.pntd.0011380 (PMC10266689; doi:10.1371/journal.pntd.0011380)
Supplement: S1 Table — All sequences were obtained from Vector Base (https://www.vectorbase.org/) and primers were synthesized by Exxtend. T7 promoter sequence is underlined. (DOCX) [file pntd.0011380.s003.docx]

**Table S1: Genes and primers List.** All sequences were obtained from *Vector Base* (<https://www.vectorbase.org/>) and primers were synthesized by Exxtend. T7 promoter sequence is underlined.

| **GENE** | **VECTOR**  **BASE** | **PRIMER SEQUENCE (5’-3’)** | **AMPLICON**  **(bp)** | **PRIMER EFFICIENCY** |
| --- | --- | --- | --- | --- |
| **18S** | RPRC017412 | FOR: TCGGCCAACAAAAGTACACA  REV: TGTCGGTGTAACTGGCATGT | 105 | **Slope:** -3,542  **Y-inter:** 11,523  **R2:** 0,991  **Efficiency %:** 91,557 |
| **Prosα6 (qPCR)** | RPRC007220 | FOR: CTTAGTCGCCACATGAGAACTG  REV: CGTACCTTTGCGTAGAATGTTG | 121 | **Slope:** -3,085  **Y-inter:** 24,769  **R2:** 0,97  **Efficiency %:** 110,956 |
| **Prosα6 (dsRNA)** |  | FOR: TAATACGACTCACTATAGGGTACTCGGAGTAGGCCTATTGGTTG  REV: TAATACGACTCACTATAGGGTACTGGTGTCCATTGCCACTTGTG | 460 | - |
| **ATG1**  **(ULK1)** | RPRC009624 | FOR: GTACTGGCGTTGAGTGAATGTG  REV: CATTGCTTACTGTAGGCGATGG | 166 | (1) |
| **ATG3** | RPRC008742 | FOR: CCAGAAGAATTTGTTGCTGCG  REV: CGATTGATAGACCCCGACGATCC | 203 | (2) |
| **ATG6 (Beclin1)** | RPRC006439 | FOR: CCGCTCCTGTAGACTGGTC  REV: GCCACCATCGCAGCATCAAATTTTG | 226 | (3) |
| **ATG8 (LC3)** | RPRC014434 | FOR: GAACAATGTAATCCCACCGACAAG  REV: CCATAGACATTTTCATCACTATACGC | 108 | (4) |
| **p62** | Non annotated | FOR: AATGACGTTTTGAAGGCGGG  REV: TTGCTCGATGTTGCCGTTTT | 225 | **Slope:** -3,486  **Y-inter:** 26,924  **R2:** 0,982  **Efficiency %:** 93.592 |
| **E1** | RPRC003935 | FOR: GCTCCAAAACTCAAGTACTATGAG  REV: ACAAGTGCTCTAACATGCGGTTCA | 263 | (5) |
| **E2.1** | RPRC007710 | FOR: AGAAGCGGCTGAAGTTTTGC  REV: GAACGCGGACAATCGCAGTA | 156 | (5) |
| **E2.2** | RPRC006315 | FOR: TTACCCATCCAGTCCTCCAAAA  REV: CTCTTTTCATACTCAAGCCTGT | 240 | (5) |
| **PoliUBQ** | RPRC012247 | FOR: AAGGAATTCCACACCAACA  REV: GAGGGCTCAACTTCAAGAGT | 188 | (5) |
| **BiP1** | RPRC009759 | FOR: GAGCGCAACGCTAGAATACC  REV: TGGCATCCAGATCGAATGTA | 205 | (6) |
| **BiP2** | RPRC006365 | FOR: GGTATCCCCCAGATTGAGGT  REV: TGCGTTTCTAGCATTGATCG | 216 | (6) |
| **BiP3** | RPRC010252 | FOR: AGGTGGTGGAACCTTTGATG  REV: GATGATAATGCTCGCTTGGC | 228 | (6) |
| **BiP4** | RPRC004310 | FOR: CTTATGGAGCTGCTGTGCAA  REV: CTCCGGGTTGGTTATCTGAA | 205 | (6) |
| **BiP5 (GRP78)** | RPRC013386 | FOR: CTAACACCGGCAACACCTTT  REV: ACTCTCCGCTGTTTCCTTCA | 213 | (6) |
| **PDI2** | RPRC004119 | FOR: TCACTCTTGCCAAGGTCGAT  REV: TTCGCACTTGGACCAACTTG | 181 | (6) |
| **PDI4** | RPRC010401 | FOR: TTTCACTGGAGGCCTAGACG  REV: GGATAGAGTGCAGCCGTTTG | 208 | (6) |
| **PDI5** | RPRC002610 | FOR: GGAATCAATCTGCACCGAGT  REV: CGCACAACAGATGGAGTAGC | 179 | (6) |
| **VgR** | RPRC000551 | FOR: TTACAGCATATCGTCCTCCG  REV: GGGAGAACGGCAGACATTGT | 216 | **Slope:** -3,247  **Y-inter:** 25,455  **R2:** 0,98  **Efficiency %:** 103,211 |

1. Bomfim L, Ramos I. Deficiency of ULK1/ATG1 in the follicle cells disturbs ER homeostasis and causes defective chorion deposition in the vector Rhodnius prolixus. FASEB J Off Publ Fed Am Soc Exp Biol. 2020 Oct;34(10):13561–72.

2. Anna, Santos; Isabela R. ATG3 is important for the chorion ultrastructure during oogenesis in the insect vector Rhodnius prolixus. Forntiers Physiol. 2021;In press.

3. Vieira PH, Bomfim L, Atella GC, Masuda H, Ramos I. Silencing of RpATG6 impaired the yolk accumulation and the biogenesis of the yolk organelles in the insect vector R. prolixus. PLoS Negl Trop Dis. 2018;12(5):1–19.

4. Pereira J, Diogo C, Fonseca A, Bomfim L, Cardoso P, Santos A, et al. Silencing of RpATG8 impairs the biogenesis of maternal autophagosomes in vitellogenic oocytes, but does not interrupt follicular atresia in the insect vector Rhodnius prolixus. PLoS Negl Trop Dis. 2020;14(1):e0008012.

5. Pereira J, Dias R, Ramos I. Knockdown of E1- and E2-ubiquitin enzymes triggers defective chorion biogenesis and modulation of autophagy-related genes in the follicle cells of the vector Rhodnius prolixus. J Cell Physiol. 2022;1:12.

6. Rios T, Bomfim L, Ramos I. The transition from vitellogenesis to choriogenesis triggers the downregulation of the UPR sensors IRE1 and PERK and alterations in the ER architecture in the follicle cells of the vector Rhodnius prolixus. Cell Tissue Res. 2022;387(1):63–74.
