# Supplementary material for: Silencing of the 20S proteasomal subunit-α6 triggers full oogenesis arrest and increased mRNA levels of the selective autophagy adaptor protein p62/SQSTM1 in the ovary of the vector Rhodnius prolixus
Source: PLoS Negl Trop Dis. 2023 Jun 2;17(6):e0011380. doi: 10.1371/journal.pntd.0011380 (PMC10266689; doi:10.1371/journal.pntd.0011380)
Supplement: S3 Table — (PDF) [file pntd.0011380.s005.pdf]

| Proteasome Subunit | VectorBase ID | Tree color | RPKM |
|---|---|---|---|
| Alpha subunit type-1 | RPRC007074 | | 210 |
| Alpha subunit type-2 | RPRC006558/RPRC009301/ RPRC003608 | | nd/nd/130 |
| Alpha subunit type-3 | RPRC006554 | | 114 |
| Alpha subunit type-4 | RPRC008753 | | 150 |
| Alpha subunit type-5 | RPRC006893 | | 138 |
| Alpha subunit type-6 | RPRC007220 | | 221 |
| Alpha subunit type-7 | RPRC002884 | | 197 |
| Beta subunit type-1 | RPRC003478 | | 113 |
| Beta subunit type-2 | RPRC014173 | | 143 |
| Beta subunit type-3 | RPRC005067 | | 142 |
| Beta subunit type-4 | RPRC014045 | | 102 |
| Beta subunit type-5 | RPRC001372 | | nd |
| Beta subunit type-6 | RPRC007007 | | 121 |
| Beta subunit type-7 | RPRC013990 | | 157 |
| Uncharacterized | RPRC009302 | | nd |

**Table S3: 20S proteasome proteins identified in *R. prolixus* and their RPKM in the mature egg transcriptome.**
