# Supplementary material for: Silencing of the 20S proteasomal subunit-α6 triggers full oogenesis arrest and increased mRNA levels of the selective autophagy adaptor protein p62/SQSTM1 in the ovary of the vector Rhodnius prolixus
Source: PLoS Negl Trop Dis. 2023 Jun 2;17(6):e0011380. doi: 10.1371/journal.pntd.0011380 (PMC10266689; doi:10.1371/journal.pntd.0011380)
Supplement: Attachment — Submitted filename: Alpha6_authors answers_R1.docx [file pntd.0011380.s007.docx]

**R1- Manuscript # PNTD-D-23-00114**

**Reviewer #1:** General comments:

In the work of Faria-Reis and co-workers entitled “Silencing of the 20S proteasomal subunit-1 α6 triggers full oogenesis arrest and modulation of the selective autophagy adaptor protein p62/SQSTM1 in the ovary of the vector Rhodnius prolixus”, the authors have studied the role of a proteasomal subunit in the oogenesis of the Chagas disease vector and model insect R. prolixus. To do so, the authors employed a series of bioinformatics, biochemistry as well as cell and molecular biology approaches. The work deals with a relevant issue, since the basic knowledge obtained could be of use to identify molecular targets for insect control. The manuscript is well organized and written, and their objectives as well as the experimental design are clear and straightforward. The findings are compelling and the conclusions match the reach of findings. Some minor suggestions are included above.

**Author´s answer:** We thank the reviewer for the time to read our manuscript and for the positive recommendation.

Specific comments

- Page 6, lines 110-111: "Daphnia magna" is misspelled. Please correct.

**Author´s answer:** Corrected.

- Page 9, line 295: In order to facilitate the comprehension for a wider audience of readers, please state what “RPKM” stands for.

**Author´s answer: “**reads per kilobase of transcript per million reads”. Added.

- Page 10, lines 305-317: Why some assessments are conducted in the midgut but some others are not? Please justify.

**Author´s answer:** Most proteasomal activity kits detect chymotrypsin like activity. We avoided measuring proteasomal activity in midgut extracts due to the likely contamination of digestive chymotrypsin like enzymes secreted by this tissue.

- Page 10, lines 321-327: Why did the authors not weight the midguts? That would have prevented to rely on indirect measurements such as the weight of the whole insect (Fig. 4B) or subjective ones such as morphology (Fig. 4C).

**Author´s answer:** We agree that this approach is not the best way to access digestion. To address this, we have performed new experiments of midgut protein quantifications at different days after feeding and added this to the revised manuscript. Interestingly, the data pointed to a more robust phenotype of impaired blood meal digestion. The new data was described as Fig 4B and mentioned in the discussion:

“The fact that we discovered the same levels of total proteins in the hemolymphs of control and silenced insects is another result that merits attention. This finding suggests that the steady state availability of yolk proteins to be incorporated by the developing oocytes is comparable in both circumstances, even though blood digestion was also partially compromised in silenced insects.”

- Page 10, line 353: The phrase “To investigate the fat body vitellogenesis capacity,” when related to the proteins observed in the hemolymph is not entirely accurate, since the result of the proteins circulating in the hemolymph is not only dependent on the fat body. It also results from the dynamic process of the protein uptake by the oocytes and other organs as well as from the different mechanisms of protein degradation. In fact, this point is well covered in the discussion. Please rewrite.

**Author´s answer:** Agreed and done. The new text in the revised manuscript is copied below:

“Furthermore, the overall hemolymph protein levels (Fig 4K) and profile, as seen by SDS-PAGE (Fig 4L, arrowheads point to the major circulating yolk protein vitellogenin [75]), were similar in control and Prosα6-silenced females, demonstrating that the availability of yolk proteins to be collected into the oocytes during vitellogenesis was not affected in the silenced insects.”

- Since there seems to be no differences in the offer of hemolymph proteins between silenced and control individuals, have the authors studied the uptake of yolk proteins in ovarian follicle? Did they measure the expression of vitellogenin receptor in the ovary? Even if they have preliminary data, this could be relevant to generate new hypothesis and/or to enrich the discussion.

**Author´s answer:** Agreed. We carried out the indicated experiment and assessed VgR expression in control and silenced ovaries at various days after feeding. We found that, in control insects, VgR expression presents a tendency of elevation in the ovaries on days 5 and 7 after feeding. Interestingly, in silenced ovaries, VgR expression presented a similar profile but displaying higher expression levels than control samples on days 5, 7 and 10 after feeding. This information was incorporated and discussed in the revised paper as Fig 5F. Copy of the text underneath:

Results:

“Because the uptake of the main yolk protein Vg by the oocytes is carried out by the vitellogenin receptor (VgR), we asked whether or not its expression was altered in Prosα6-silenced ovaries. We found that in control insects VgR expression presents a tendency of elevation in the ovaries on days 5 and 7 after feeding. Interestingly, in silenced ovaries, VgR expression presented a similar profile but displaying higher expression levels than control samples on days 5, 7 and 10 after feeding (Fig 5F).”

Discussion:

“Interestingly, we found that that the VgR is more expressed in Prosα6-silenced ovaries when compared to controls. Although specific conclusions cannot be drawn from these data, the greater VgR levels in the silenced samples can be the result of a transcriptional compensatory mechanism in response to the delayed Vg absorption and oocyte maturation. It is possible that in the absence of endocytosis-receptor recycling, VgR resides in the plasma membrane, causing it to undergo programmed destruction, which in turn activates a transcriptional upregulation [10,94–96].”

**Reviewer #2:**

Faria-Reis et al. identified genes that encode α and β subunits of the 20S proteasome in the genome of R. prolixus and, taking into account another MS, they concluded that those genes are maternally accumulated in mature oocytes. Then, they downregulated the transcript expression of one of the 20S proteasome subunits (Prosα6) as a tool to suppress 20S proteasomal activity. The results demonstrated that the downregulation of Prosα6 triggers full oogenesis arrest as well as changes in the transcript expression of adaptor protein p62/SQSTM1. Overall, Faria-Reis et al. show that proteasome activity is especially important for signals to initiate oogenesis. I consider this work contains very interesting results; however, it is important to address several points/observations to improve the understanding of the experiments and conclusions.

**Author´s answer:** We thank the reviewer for the time to read our manuscript and for the positive recommendation.

1) Title: here, the authors mention “…modulation of the selective autophagy adaptor protein p62/SQSTM1…”, but the truth is that it was just a finding from a qPCR; in the present MS, the authors only show transcript level variations but they didn’t investigate the protein or the functional involvement of p62/SQSTM1. Thus, I consider a little ambitious to leave it in the title, for that case, a deeper study should be done.

**Author´s answer:** Agreed. The title was changed to: “Silencing of the 20S proteasomal subunit-α6 triggers full oogenesis arrest and increased mRNA levels of the selective autophagy adaptor protein p62/SQSTM1 in the ovary of the vector *Rhodnius prolixus”*

2) Line 26: I don’t think that “genes are maternally accumulated in the transcriptome”, perhaps, transcripts accumulated into mature oocytes.

**Author´s answer:** Agreed. The rephrased text in the revised manuscript now reads:

“Here, we used bioinformatics to identify the genes that encode the seven α and β subunits of the 20S proteasome in the genome of *R. prolixus* and learned that those transcripts are accumulated into mature oocytes.”

3) General speaking, the introduction is well written, there is a nice description about the UPS and the proteolytic complex 26S proteasome. I would like to read why the authors chose to study 20S and not 19S sub-complex.

**Author´s answer:** We chose to disturb the 20S complex because it is the catalytic complex, and we wanted to study the role of proteasomal activity during oogenesis.

Also, I really believe that an introduction on Rhodnius reproductive physiology is missing since the entire MS is based on events that happen over a female reproductive cycle.

**Author´s answer:** Agreed. The following text was added to our introduction:

“The reproductive systems of female insects have been extensively studied due to their potential as targets for insect control. Basically, the maturing oocytes internalize yolk proteins via receptor-mediated endocytosis after the fat body produces and secretes vitellogenin (Vg) and other precursors of yolk proteins, and this is a crucial phase in the female reproduction process in most insects (Roy et al., 2018; Lange et al., 2022). The adult female reproductive system of *R. prolixus* is composed of two ovaries, each containing 7 telotrophic ovarioles. Oogenesis has three phases known as pre-vitellogenesis, vitellogenesis and choriogenesis. In the vitellarium, pre-vitellogenic and vitellogenic oocytes are connected to the nurse cells in the tropharium by a nutritive cord, which allows the transport of macromolecules, mRNA, and protein to the developing oocytes (Vanderberg, 1963). Vitellogenin (Vg), the main yolk protein precursor, is mostly provided by the fat body, but it is also synthesized within the follicle cells and delivered to the oocyte in the late phase of growth (Oliveira et al., 1986; Melo et al., 2000). Once vitellogenesis is complete, the trophic cord is severed, the chorion is synthesized and secreted, and the mature egg becomes ready to be fertilized and laid in the environment.”

4) Line 52: reference 2 is not correctly written in “References” section

**Author´s answer:** Corrected. The corrected reference is now number 6.

5) Line 68: double space

**Author´s answer:** Done.

6) Line 123: How the authors know that females were mated? Why the authors prefer females from the second or third blood feeding for experiments? Please, add to the MS your responses. Also, indicate how much the females are fed because for a full egg production it must be a minimum of times by weight.

**Author´s answer:** All females used in this work were obtained from our insectarium where the insects are fed for the first time (as adult insects) in live-rabbit blood 14 days after the 5th instar nymph to adult ecdysis. After the first blood feeding, the insects (male and females) are kept back together to mate and generate the eggs that will hatch as first instar nymphs to maintain the insectarium. We know that the females were mated because we do monitor their oviposition rates and F1 eclosion rates during this cycle (virgin females do not lay many eggs, and they do not hatch because they are not fertilized). After this first blood feeding, all adult females are fed every 21 days and only fully gorged insects (allowed to feed at free demand, usually gaining 6-7 times the insect’s initial body weight in 20-30 min) are used for the experiments. Thus, females of the second or third blood feeding were used, and they are highly synchronized regarding blood feeding, digestion, and oviposition.

7) Line 124: it seems that all dissections were always carried out on day 7 post blood meal and not on different days. Also, why did the authors choose 7 d PBM for experiments?

**Author´s answer:** In our hands, 7 days after the blood meal is the first time point when the dissection of the vitellogenic ovaries renders the observation of the first batch of chorionated (mature) oocytes, so we know that the first cycle of oocyte development is terminated, and the ovary samples include developing and fully grown oocytes.

8) Line 135: ovarian follicles

**Author´s answer:** Corrected.

9) Line 136: …in phosphate-buffered saline (PBS, 137 mM NaCl, 2.7 137 mM KCl, 10 mM Na2HPO4, and 1.8 mM KH2PO4, pH 7.4) using…

**Author´s answer:** Done.

10) Lines 141, 142 and others: according to Author_name et al. [X]

**Author´s answer:** Done.

11) Line 144: Total RNA

**Author´s answer:** Done.

12) Line 145 TRIzol

**Author´s answer:** Done.

13) Lines 156 and 160: It seems that the same primers were used to regular PCR and qPCR but using a different annealing temperature; was the primer efficiency tested with both temperatures?

**Author´s answer:** The regular PCRs were used only as a first test to access primer’s functionality. All experiments including primers efficiencies, melting curve analysis, blank reactions, etc were performed using the qPCR protocol (with 60oC annealing temperature).

14) Line 157: According to MIQE, the final acronym ‘RT-qPCR’ is used for reverse transcription quantitative real-time PCR.

**Author´s answer:** Corrected.

15) Line 165: Add reference for the use of dCt method and give the complete name for 18S the first time that it is named.

**Author´s answer:** 18S ribosomal RNA (18S) and the reference were added.

(Livak and Schmittgen, 2001)

16) Line 168: the word “invariant”, is not appropriate, is not the correct expression for this case.
**Author´s answer:** The word “invariant” was changed to “stable”

17) Check when to use Table S1 or supplementary Table 1

**Author´s answer:** Done.

18) The RNAi experiments were done lack an off-target control. The authors should mention this point in the MS.

**Author´s answer:** The following sentence was added to the revised discussion:

“Finally, it's crucial to note that even though we performed in silico tests for potential off targets and found no significant results, it's still impossible to entirely rule out off target effects that could partially explain the reported phenotypes.”

19) Line 184: How the absence of cross-reactivity was tested? Please, add as supplementary Figure a complete image of western blot, showing controls and molecular weight of the protein of interest.

**Author´s answer:** The pre-immune serum of the immunized rabbit was tested and showed no reactivity in the expected molecular weight of Prosα6 (~30kDa). Also, the final anti-Prosα6 antiserum presented an ELISA title of 1:512.000 against the generated peptide (as tested by Gene Script). The complete blots were added as Fig S2.

20) Line 190: Was the Lowry method performed without adaptations? It is a method published in 1951. Did the authors use a kit or a microtechnique? Please, clarify in the MS

**Author´s answer:** A micro technique adapted to a 96 well plate and a standard curve ranging from 1-5 µg of bovine serum albumin (BSA). This was added to the revised manuscript.

21) Line 195: The extraction buffer usually contains detergents to optimize the efficiency of extraction of soluble proteins. It is weird that in this case, the extraction buffer is just PBS and the authors didn’t mention any centrifugation step; is that correct?

**Author´s answer:** Yes, the usual buffers in the most cited protocols to isolate functional proteasome particles do not include any detergent (Li et al., 2015; Wang et al., 2021). So, we assumed that there was no need to add detergents to extract proteasome proteins in the ovaries for the immunoblottings. Regarding centrifugation steps, since we do not know much about the compartmentalization of proteasome particles in the oocytes, we avoided those steps to make sure that a differential centrifugation step would not wash away the particles of interest.

22) Line 202: “…the membranes were revealed…”

**Author´s answer:** Done.

23) Line 218: Adult female reproductive system of R. prolixus is composed of 2 ovaries; are the authors considering just one ovary or both from an insect? Please, clarify it throughout the MS

**Author´s answer:** Both from an insect. Info added to Methods>Dissection of organs and ovarian follicles.

24) For each experiment, did the authors use a pool of tissues or an individual tissue? Please, check it throughout the MS and add the information where necessary. As it is now, it’s not clear. Also, “N” usually refers to the population size and “n” usually refers to the sample size, how are the authors using in this case?

**Author´s answer:** Corrected to n (lowercase). We are referring to sample size. The captions now indicate when pools of tissues were used.

25) Line 242: Was the hemolymph centrifugated to remove hemocytes?

**Author´s answer:** No. The experiments made use of unmodified hemolymph samples.

26) Line 246: Sentences should never begin a numeral; instead, the authors should try to reword the sentence.

**Author´s answer:** Rephrased to “Freshly laid (0-24h) eggs…”

27) Line 255: What is the basis for selecting t-test or chi squared test in this MS?

**Author´s answer:** T-tests and chi-square tests can both evaluate differences between two groups. However, a t test must be utilized when there are two groups in dependent quantitative variable. In cases where there are two categorical variables, a chi-square test of independence was applied (Bliss, 1967).

28) Line 272: where the transcriptome data is deposited? Also, you should discuss taking into account that the data from that transcriptome is from unmated females and you are using mated ones.

**Author´s answer:** (Coelho et al., 2021). We contacted the authors and found that the transcriptome raw data was not yet deposited in any database but it is available upon request. The following text was added to our results:

“Although the transcriptome was performed using unmated females, this finding indicates that the majority of the 20S proteasome's functional components are maternally accumulated in the oocytes, presuming a significant role for proteasomal degradation during oogenesis and/or early development.”

29) Line 296: abbreviation Prosα6 was already defined above.

**Author´s answer:** Corrected.

30) Line 291-295: this phrase means the same that was written in lines 307-309.

**Author´s answer:** Lines 307-309 were rephrased to: “To learn more about how vitellogenesis and oogenesis are impacted by the disturbance of proteasomal activity”.

31) Line 322: the low survival rate stolen my attention; how old these insects are?

**Author´s answer:** The insects are adults from the second or third blood feeding (please see answer #6), so the insects are not old. However, in our hands, despite our efforts to maintain our insectarium under strict conditions of blood feeding cycles, temperature, humidity, and light and dark cycles, we recurrently observe a high seasonal variation on the average lifespan of our insects. These experiments were conducted in a season when the average lifespan was lower than average. It is important to note, however, that all of our control insects do not display any changes on their average rates of blood digestion, oviposition and eclosion, regardless of their lower longevity.

32) Lines 323-324: can the authors add a reference for this assumption? In my opinion, this assumption should be taken with caution because when a female 7 d PBM is weighed, the values obtained are not only a representation of the MG content but also of eggs inside of those insects.

Also, the truth is that according to Fig 4B, it seems to me that the weight is significatively higher in dsProsα6. Could the authors show the stat for each day?

**Author´s answer:** We agree that this approach is not the best way to access digestion. To address this, we have performed new experiments of midgut protein quantifications at different days after feeding and replaced this data in the revised manuscript. Interestingly, the new data pointed to a more robust phenotype of impaired blood meal digestion. The new data was described as Fig 4B and mentioned in the discussion:

“The fact that we discovered the same levels of total proteins in the hemolymphs of control and silenced insects is another result that merits attention. This finding suggests that the steady state availability of yolk proteins to be incorporated by the developing oocytes is comparable in both circumstances, even though blood digestion was also partially compromised in silenced insects.”

33) Lines 345-346: the authors justify the analysis of UPR because of changes in the UPS and autophagy but the silencing of Prosα6 didn’t trigger any change. Please, rewrite this sentence.

**Author´s answer:** Done, the beginning of the first sentence was deleted.

34) Line 350: and BIP2? Also, sometimes it is written BiP and other times BIP.

**Author´s answer:** BiP2 was mentioned and BiP/BIP was corrected.

“We also examined the expression of the ER chaperones HSP70/BiPs and PDIs, which are recognized effectors induced by the activation of the UPR. Interestingly, BiP5/GRP78 displayed a 7-fold induction, consistent with an activation of the UPR. On the other hand, BiP2 expression was strongly inhibited, while all the other isoforms of BiPs and PDIs did not exhibit any changes in expression in Pros6-silenced fat bodies when compared to control samples (Fig 4J).”

35) Lines 353-355: Please, rewrite this sentence, it is not grammatically well written.

**Author´s answer:** Done. The new text now reads:

“Furthermore, the overall hemolymph protein levels (Fig 4K) and profile as seen by SDS-PAGE (Fig 4L, arrowheads point to the major circulating yolk protein vitellogenin (Vg) (Masuda and Oliveira, 1985)), were similar in control and Prosα6-silenced females, demonstrating that the availability of yolk proteins to be collected into the oocytes during vitellogenesis was not affected in the silenced insects.”

36) Line 376: It should be clarified when the authors draw conclusions or hypothesis on results that show non-statistically significant differences.

**Author´s answer:** Done. The text now mentions tendencies when the data was not statistically significant.

37) Line 408: Please, add a reference for this statement.

**Author´s answer:** References added: (Oliveira et al., 1986, 1995)

38) Line 418: in line 350 it is written 7-fold

**Author´s answer:** Corrected to 7-fold.

39) Line 411: The authors could discuss in this part about the transcript expression for Prosα6 being higher in ovaries with respect to other tissues. Also, to draw conclusion on Prosα6 maternally accumulated, please, discuss about insect oocytes pointing out that they are transcriptionally silent during vitellogenesis.

**Author´s answer:** Agreed. The new text was added and copied here:

“It is interesting to point that during oogenesis the developing oocyte is undergoing meiosis, and its pronucleus is mostly transcriptionally inactive. Most of its accumulated mRNA is maternally derived synthesized by the nurse cells. In a meroistic-telotrophic type of ovary, such as in *R. prolixus*, the accessory nurse cells are located in the tropharium being connected to the oocytes through cytoplasmic bridges. Thus, it is probable that the high levels of Prosα6 mRNA found in the tropharium are targeted for delivery to the developing oocytes.”

40) Lines 437-438: in order to give more power to this hypothesis, the authors should show the protein content in the fat body of dsRNA-treated females. Also, it is not clear for me why if exist a feedback mechanism, circulating total proteins will be in the same amount.

**Author´s answer:** Protein content in the fat body was found to be similar between control and silenced samples.

However, we chose not to use this data since we consider this is not the best read out to access secretion rates. The flux of newly synthesized proteins to the secretory pathway is usually pretty rapid, so it is not common to observe an accumulation of the protein intended for secretion within the cell. In fact, the protein profiles of vitellogenic fat bodies of *R. prolixus* do not show accumulated Vg or RHBP, even though this organ is actively secreting high levels of these proteins to the hemolymph (Paiva-Silva et al., 2002).

41) At the oocyte membrane, VgR binds Vg and once endocytosed, VgR is recycled back from oocyte cytoplasm to the membrane, where processes as ubiquitylation can take place. Taking it into account, the authors could test VgR transcript in ovaries of dsProsα6-treated insects and elucidate if the recycled back might be working or not. It could give a good response to the oogenesis arrest lead by these insects.

**Author´s answer:** Agreed. We carried out the indicated experiment and assessed VgR expression in control and silenced ovaries at various days after feeding. We found that, in control insects, VgR expression presents a tendency of elevation in the ovaries on days 5 and 7 after feeding. Interestingly, in silenced ovaries, VgR expression presented a similar profile but displaying higher expression levels than control samples on days 5, 7 and 10 after feeding. This information incorporated and discussed in the revised paper as Fig 5F. Copy of the text underneath:

Results:

“Because the uptake of the main yolk protein Vg by the oocytes is carried out by the vitellogenin receptor (VgR), we asked whether or not its expression was altered in Prosα6-silenced ovaries. We found that in control insects VgR expression presents a tendency of elevation in the ovaries on days 5 and 7 after feeding. Interestingly, in silenced ovaries, VgR expression presented a similar profile but displaying higher expression levels than control samples on days 5, 7 and 10 after feeding (Fig 5F).”

Discussion:

“Interestingly, we found that that the VgR is more expressed in Prosα6-silenced ovaries when compared to controls. Although specific conclusions cannot be drawn from these data, the greater VgR levels in the silenced samples can be the result of a transcriptional compensatory mechanism in response to the delayed Vg absorption and oocyte maturation. It is possible that in the absence of endocytosis-receptor recycling, VgR resides in the plasma membrane, causing it to undergo programmed destruction, which in turn activates a transcriptional upregulation (Raikhel and Dhadialla, 1992; Snigirevskaya et al., 1997; Roy et al., 2018; Wu et al., 2021).”

42) Line 447: here I found a point that deserves more attention from authors. In R. prolixus juvenile hormone (JH) controls both the production of vitellogenin in the fat body and its uptake by the ovary, and ecdysteroids (Ec) control ovulation. Recently a function controlling oocyte maturation was reported, but again, the main hormone involved in promoting vitellogenesis in R. prolixus females is JH. So, it was not clear how the authors came the conclusion that Ec could be involved. Also, the references given in the MS are from mosquitos, and the truth is that today we have a lot of bibliography on the endocrine system in Rhodnius, so, please, adapt the bibliography to the model used and reinforce the involvement of Ec because it is not well understood.

**Author´s answer:** We hypothesized the involvement of Ec because their main source in the adult females are likely the ovaries, and the ovaries in silenced samples seem dysfunctional.

Anyway, we agree that a discussion including JH is necessary. The following text was added to the discussion of the revised manuscript (including Ec, JH and with *R. prolixus* references):

“It has long been known that juvenile hormone (JH) and ecdysteroids (Ec), are the main hormones that regulate female insect reproduction (Roy et al., 2018; Lange et al., 2022). In *R. prolixus*, Vg synthesis in the fat body and ovarian absorption are controlled by JH, which is released by the corpora allata and is essential for egg production (Davey, 2000; Villalobos-Sambucaro et al., 2020). JH can cross the plasma membrane and interact with a nuclear receptor and its co-activator: methoprene-tolerant (Met) and Taiman (Tai) (Wu et al., 2021; Leyria et al., 2022). It is possible that Prosα6-silencing is affecting the signals triggered by JH to disrupt fat body vitellogenesis and/or ovary Vg uptake. Experiments investigating JH titers and its receptors would be enlightening to further explore this possibility. On the other hand, while it is known that the levels of ecdysteroids in the hemolymph peak in the adult female at 5 days following a blood meal (RUEGG et al., 1981), the biochemical pathway of Ec synthesis and its effects during vitellogenesis in *R. prolixus* are still not well characterized. In adult females, the ovaries are the main source of this hemolymph ecdysteroid (Brown et al., 2008), which presumably operates by binding the heterodimeric nuclear hormone receptor made up of the ecdysone receptor (EcR) and ultra-spiracle protein (USP), as it has been described in numerous insect species (Khalid et al., 2021). In *R. prolixus*, it has been claimed that Ec from the ovaries may reduce Vg synthesis in the fat body (Garcia et al., 1979), and rather controls ovulation and oviposition (Davey, 2007). One possibility is that interference at the early stages of oogenesis (caused by the silencing of Prosα6) renders the ovary incompetent in generating Ec to maintain the signals that modulate vitellogenesis, ovulation and/or oviposition. Although the effect of Ec in the fat body vitellogenesis has not been entirely characterized in *R. prolixus*, it has been described in a number of insect taxa (Roy et al., 2018).”

43) Line 806: (C)

**Author´s answer:** Done.

44) Adapt “N” or “n” and “pool” or “individual” samples in all captions. Please, check the stat analysis throughout the MS.

**Author´s answer:** All info was added to the captions. Basically, for qPCRs we used pools of 2-3 insects per experiment. For the other experiments insects or their samples were used individually.

45) Line 812: the relative expression shown in (D) is not the dCt as state in M&M. Please, indicate in the figure, M&M and caption that it is a percentage.

**Author´s answer:** Done.

46) What the criteria was to choose FB and OV for same test (Fig 2 and Fig 3F) and OV, FB and MG in others (Fig 3B and D)?

**Author´s answer:** Most proteasomal activity kits detect chymotrypsin like activity. We avoided measuring proteasomal activity in midgut extracts due to the likely contamination of digestive chymotrypsin like enzymes secreted by this tissue.

47) Please, indicate molecular weight and add as supplementary figure the complete blot

**Author´s answer:** Done. The complete blot was added as Fig S2. Please see answer #19.

48) Line 813: indicate in M&M how the densitometric quantification was done.

**Author´s answer:** Done. Text added to M&M>Immunoblottings of the revised manuscript: “The intensity of the bands was analyzed by densitometry with ImageJ software version 1.50i, with background corrections.”

49) Line 822: weighed

**Author´s answer:** Corrected.

50) Line 827: what does “2 experiments” mean? n=2?

**Author´s answer:** Yes. It was rephrased and corrected in the caption to n=2.

51) Line 835: Total protein in hemolymph from control and Prosα6-silenced females measured by…

**Author´s answer:** Done.

52) Line 841: it seems that was by 3 weeks.

**Author´s answer:** Corrected.

53) Line 842: on the graph (Fig 5B) there are just 16 points, does it mean n=16? Because in the captions is written N=45. Also, change to ****

**Author´s answer:** Yes, this was a mistake. We apologize. It was corrected to n=16.

54) The stat in Fig 5A is not clear. Also, add more numbers to the y and x axes to make it easier to interpret.

**Author´s answer:** Done. Oviposition is statistically different between control and silenced samples at the time points indicated by the bar. More numbers were added to the axes.

55) Line 843: It stolen my attention that there are N=45 for both groups since the eggs laid/ Prosα6-silenced females is very low; is it correct?

**Author´s answer:** The n=45 was a mistake and corrected to n=16-20. We apologize for this. The graph shows the mean+-SEM of the number of eggs per female from 16-20 insects individually.

56) Line 844: It is not only ovaries but also the calyx and oviducts; perhaps, it is more correct to write “reproductive system.

**Author´s answer:** Done. The text now reads: “Representative image of dissected reproductive systems (ovaries, calyx and oviducts) from control and Prosα6-silenced females…”

57) Schematic diagram: in general, it is a nice way to draw conclusions, just modify according to the revision. Also, Prosα6 is not inhibited but its transcript is downregulated.

**Author´s answer:** Done. The diagram was altered according to the revision.

58) Check for µL or µL.

**Author´s answer:** Done.
